# Disaster Risk Perception, Sense of Pace, Evacuation Willingness, and Relocation Willingness of Rural Households in Earthquake-Stricken Areas: Evidence from Sichuan Province, China

**DOI:** 10.3390/ijerph17020602

**Published:** 2020-01-17

**Authors:** Dingde Xu, Chen Qing, Xin Deng, Zhuolin Yong, Wenfeng Zhou, Zhixing Ma

**Affiliations:** 1 Sichuan Center for Rural Development Research, College of Management of Sichuan Agricultural University, Chengdu 611130, China; 2 College of Management of Sichuan Agricultural University, Chengdu 611130, China; qingchen@stu.sicau.edu.cn (C.Q.); zhuolinyong@stu.sicau.edu.cn (Z.Y.); wenfengzhou@stu.sicau.edu.cn (W.Z.); zhixingma@stu.sicau.edu.cn (Z.M.); 3 College of Economics of Sichuan Agricultural University, Chengdu 611130, China; dengxin@sicau.edu.cn

**Keywords:** risk perception, sense of place, evacuation willingness, relocation willingness, earthquake, China

## Abstract

Based on survey data from 327 rural households in the areas affected by the Wenchuan Earthquake and Lushan Earthquake in Sichuan Province, this study systematically analyzed disaster risk perception, sense of place, evacuation willingness, and relocation willingness among residents in these earthquake-stricken areas. Further, this study constructed an ordinal logistic regression analysis to probe the correlations between residents’ disaster risk perception or sense of place and evacuation willingness and relocation willingness, respectively. The results showed that (1) faced with the threat of earthquake disasters, residents have a strong willingness to evacuate and relocate. Specifically, 93% and 78% of the residents in the Wenchuan Earthquake and Lushan Earthquake areas were willing to evacuate and relocate, respectively, whereas 4% and 17% of the residents were unwilling to evacuate and relocate, respectively. (2) Place dependence and the severity of disaster occurrence were significantly positively correlated with residents’ evacuation willingness, while the interaction term between place dependence and the severity of disaster occurrence was negatively related to residents’ evacuation willingness. Specifically, when everything else remains constant, every one-unit increase in place dependence and severity corresponds to increases in the odds of willingness to evacuate by factors of 0.042 and 0.051, respectively; every one-unit increase in place dependence × severity corresponds to a decrease in the odds of willingness to evacuation by a factor of 0.004. (3) Place identity was significantly negatively correlated with residents’ relocation willingness, while place dependence and severity of disaster occurrence were positively related to residents’ relocation willingness. The interaction term between place dependence and the severity of disaster occurrence as well as the interaction term between place identity and severity of disaster occurrence were significantly negatively correlated with residents’ relocation willingness. Specifically, every one-unit increase in place identity corresponds to a decrease in the odds of willingness to relocate by a factor of 0.034, while every one-unit increase in place dependence and severity corresponds to increases in the odds of willingness to relocate by factors of 0.041 and 0.028, respectively, and every one-unit increase in place dependence × severity and place identity × severity corresponds to decreases in the odds of willingness to relocate by factors of 0.003 and 0.003, respectively.

## 1. Introduction

Over the past 20 years, with the movement of geological plates, earthquake disasters have occurred frequently around the world, causing serious losses to human life and property [1,2,3,4]. Statistics indicate that between 2000 and 2018, 830,400 people were affected and 87,900 people were killed by earthquake disasters worldwide [5]. China is a mountainous country and has suffered from frequent earthquake disasters in recent years, leading to disastrous losses to human life. Since 2008, Sichuan Province in China has experienced three earthquakes with magnitudes of 7 or above, including the 5•12 Wenchuan Earthquake, the 4•20 Lushan Earthquake, and the 8•8 Jiuzhaigou Earthquake, each of which has had a significant impact on local residents. The three great earthquakes are reported to have resulted in a total of 459,900 casualties and direct economic losses of 932 billion Yuan [6]. Due to the suddenness and seriousness of earthquake disasters, the construction of resilient disaster prevention systems in earthquake-stricken areas has gradually attracted the attention of the political and academic leaders and has become a focus of research in the field of disaster risk management. However, the existing studies of earthquake disaster risk management primarily originate from developed countries (e.g., [7,8,9,10]); there are relatively few studies of earthquake disaster risk management in China, a developing country. Therefore, it is critical to perform this research in the Chinese context [2,11].

Effective disaster preparedness can enhance the disaster prevention capabilities of families at risk of facing a disaster and can reduce the impact of such disasters on these families [12,13,14,15,16]. For example, the study by Godschalk et al. [17] in the United States found that every $1 increase in disaster prevention investment can help residents reduce losses by $4. Among the common disaster preparedness measures, effective evacuation and reasonable relocation in the event of a disaster are the most effective measures to ensure the safety of residents’ lives and properties. Existing studies in this field have focused on residents’ evacuation willingness or evacuation behavior (e.g., [18,19,20,21,22,23]); however, few studies have focused on residents’ relocation willingness or relocation behavior [24]. Evacuation and relocation are two different concepts. Specifically, evacuation involves residents temporarily leaving their homes with the intention to return after the disaster, whereas relocation usually involves residents moving out of their homes completely and relocating to another safer place. Unlike developed countries, which are sparsely populated, 70% of China’s mountainous areas are inhabited by 45% of the population [25,26], and many residents live in areas at threat of disasters [27,28,29]. In order to cope with disasters, residents need to be ready to evacuate at any time. When the local government has sufficient financial resources, or the residents themselves have the ability to move out, several residents will choose to relocate. In China, and in many developing countries around the world, mountain settlements are facing serious disaster threats and must be relocated. Therefore, it is necessary to conduct comparative research on evacuation willingness and relocation willingness of residents in disaster threat areas.

Evacuation and relocation are effective means to reduce the impact of disasters on residents, and the mechanisms driving these behaviors are a focus of academic research. The existing studies have examined several types of indicators that may affect residents’ disaster preparedness (including the willingness for evacuation and relocation as well as the actual behaviors of evacuation and relocation), including individual and family socio-economic characteristics of residents (e.g., [18,30,31]), disaster information sources (e.g., [19,22,32]), disaster experiences (e.g., [24,33]), and disaster risk perception (e.g., [21,34,35,36,37,38,39,40,41,42]). Among these indicators, sense of place is a factor that has been rarely considered in the existing research [24,43,44]. In the limited available quantitative research, the correlation between residents’ sense of place and disaster preparedness is not consistent. For instance, Paton [44] reported that community attachment was not significantly correlated with disaster preparedness, while Xu et al. [24] found that place identity and place dependence of residents in landslide threat areas were significantly negatively related to relocation willingness. Similar variation in findings is also evident for individual and family socio-economic characteristics of residents, disaster risk perception, and other commonly used indicators. The differing results among studies are likely due to the different types of disasters and the different social, economic, and cultural environments of the regions under study [24,33]. For example, Siegrist and Gutscher [45] found that residents’ disaster risk perception was not significantly correlated with disaster avoidance behavior, while Mcneill et al. [46] reported that the possibility and severity of disaster occurrence were significantly positively related to residents’ avoidance behavior. Xu et al. [24] found that the possibility and threat of disaster occurrence were significantly positively correlated with residents’ relocation willingness, and the controllability of disaster occurrence was significantly negatively related to residents’ relocation willingness. Are there significant correlations between residents’ disaster risk perception or sense of place and their evacuation willingness or relocation willingness in earthquake-stricken areas? Can sense of place increase or decrease the disaster risk perceptions of residents, thereby affecting their evacuation willingness or relocation willingness? These questions still need to be answered.

To this end, this study used survey data from 327 rural households in the earthquake-stricken areas of the Wenchuan Earthquake and the Lushan Earthquake in Sichuan Province to systematically analyze residents’ disaster risk perceptions, sense of place, evacuation willingness, and relocation willingness. Moreover, econometric models were constructed to explore the correlations between these variables to provide a further understanding of the relationships between these factors. The findings of this study can inform disaster risk management and resilient disaster prevention systems for earthquake-stricken areas. This study intends to answer the following questions:

(1) What are the characteristics of residents’ disaster risk perceptions, sense of place, evacuation willingness, and relocation willingness in earthquake-stricken areas?

(2) What are the correlations between residents’ disaster risk perception or sense of place and evacuation willingness and relocation willingness, respectively, in earthquake-stricken areas?

(3) Can residents’ sense of place in earthquake-stricken areas increase or reduce their disaster risk perceptions, thereby affecting their evacuation willingness and relocation willingness?

## 2. Data and Methods

### 2.1. Data Source

**Figure 1:**
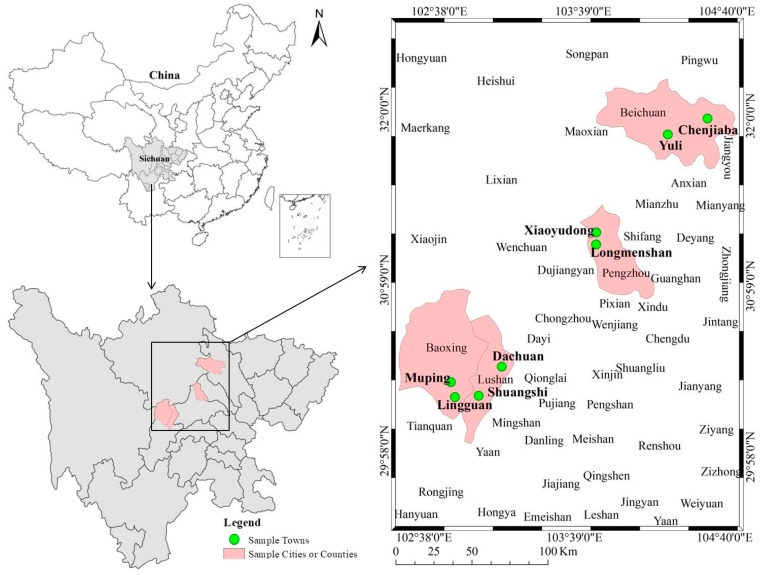
Location map of sample counties and towns.

The data used in this study are mainly from the questionnaire survey conducted by the research team in the areas stricken by the Wenchuan Earthquake and the Lushan Earthquake in July 2019, and the survey method is a one-on-one face-to-face interview. The contents of the survey mainly include rural households’ sustainable livelihoods, residents’ disaster-risk perception, residents’ disaster-avoidance behavior, the construction of a resilient disaster-prevention system in villages, and so forth. The time for each questionnaire is about one and a half hours. Some objective indicators, such as rural households’ income, are mainly adopted to inquire about the family situation of rural households by the end of 2018, whereas some subjective indicators, such as residents’ disaster risk perception, are primarily used to ask about the status of respondents when they are surveyed. In order to ensure the typicality and representativeness of the selected samples, this study chiefly adopts the stratified sampling method, which is a probability sampling technique, to determine the survey samples (Figure 1). The specific operation process is as follows:

Firstly, with regard to the selection of sample counties, this study is mainly based on the following two aspects:

1. The four sample counties should be from the areas stricken by the Wenchuan Earthquake and the Lushan Earthquake (each of the two major earthquakes involves two counties).

2. There are significant differences in economic development level between the two sample counties selected for each of the two major earthquakes.

Based on the above considerations, this study selected as sample counties Beichuan County and Pengzhou City from ten counties stricken by the Wenchuan Earthquake (Pengzhou City is a county-level city), and Baoxing County and Lushan County from six areas stricken by the Lushan Earthquake.

Secondly, after sample counties were selected, two sample townships were randomly selected from each sample county according to differences in the level of economic development within the counties and the distance from the county center and the serious-disaster situation, especially the number of threatened people. A total of eight townships were obtained.

Thirdly, after the sample townships were determined, the villages in each sample township were divided into two types in accordance with the number of threatened people in the villages, the differences in the level of economic development, the distance from the township center, and other indicators; and one village was randomly selected from each type of village as the sample village. In this way, a total of 16 villages were obtained.

Fourthly, concerning the determination of sample rural households, after the sample villages were determined, the front-line team members obtained the roster of rural households in the sample villages from village cadres, and 20–23 rural households were randomly selected from each sample village as sample rural households according to the preset random numerical tables.

Finally, 13 researchers who received strict training conducted one-on-one face-to-face interviews in the homes of rural households under the guidance of village cadres, that is, investigators asked questions one by one, and residents answered questions one by one; in the process of investigation, if there is any dispute about any question, the investigator will ask the question in different ways and finally get the answer that can reflect the objective facts. Finally, a total of 327 valid questionnaires were obtained from 16 villages of eight townships in four counties. During the data analysis, we cleaned up some questionnaires that were obviously not in line with logical common sense. Finally, the effective rate of relevant indicators used in this study was 97%.

### 2.2. Methods

#### 2.2.1. Selection and Definition of Model Variables

(1) Measurement of residents’ evacuation willingness and relocation willingness

As the dependent variables in this study, residents’ evacuation willingness and relocation willingness in earthquake-stricken areas were measured by the following two questions:

Y1: Are rural households willing to evacuate when someone in the same village evacuates? (1 = very unwilling, 2 = unwilling, 3 = average, 4 =willing, 5 = very willing).

Y2: Are rural households willing to relocate when someone in the same village relocates? (1 = very unwilling, 2 = unwilling, 3 = average, 4 =willing, 5 = very willing).

(2) Measurement of residents’ sense of place

Residents’ sense of place in earthquake-stricken areas was one of the core independent variables of this study. Based on the research of Xu et al. [24], Peng et al. [27], Jorgensen and Stedman [47], and Quinn et al. [48], this study designed questions to measure three dimensions of sense of place: place identity, place attachment, and place dependence (Table 1). The process for measurement of sense of place was as follows:

Firstly, reliability testing was conducted for the questions used to measure the three dimensions of sense of place. The results showed that the overall Cronbach’s *α* value of the nine questions was 0.77; the Cronbach’s *α* values corresponding to the internal consistency of each of the three dimensions were also above 0.6 (Table 2), indicating that the questions measuring sense of place in this study have good internal consistency and can be used for subsequent analysis.

Secondly, dimension reduction was carried out for the questions that passed the reliability test. The Kaiser–Meyer–Olkin value corresponding to the factor analysis was 0.81, and the *p*-Value of Bartlett’s test of sphericity was less than 0.001, suggesting that the factor analysis was reasonable. As shown in Table 2, the factor analysis indicated that sense of place could be further subdivided into three dimensions, namely, place identity, place attachment, and place dependence. The cumulative variance contribution rate of the three dimensions was 64.50%.

Third, based on the research of Xu et al. [24,33,49], this study used the efficiency coefficient method to convert the comprehensive scores of the three dimensions of sense of place into a percentage system. A detailed description of the efficiency coefficient method can be found in the Appendix of the study by Xu et al. [2].

(3) Measurement of residents’ disaster risk perception

Residents’ disaster risk perception in earthquake-stricken areas was the other core independent variable in this study. Based on the research by Lazo et al. [34], Riad et al. [35], Xu et al. [49], Lindell et al. [50], Lindell and Whitney [51], Lo [52], Solberg et al. [53], and Slovic [54], this study designed questions to measure two dimensions of disaster risk perception, namely, the possibility and severity of disaster occurrence (Table 3). The process for measurement of disaster risk perception was similar to that described above for sense of place. The Cronbach’s *α* values corresponding to disaster risk perception, the possibility of disaster occurrence, and the severity of disaster occurrence were 0.72, 0.70, and 0.64, respectively, and the cumulative variance contribution rate of the two dimensions was 61.92%. For brevity, the factor-loading results of the two dimensions of disaster risk perception, which were similar to Table 2, are omitted from this paper.

(4) Measurement of control variables

In order to compare the current results with similar studies, based on the research of Hoffmann and Muttarak [12], Adeola [18], Xu et al. [24,33], Bateman and Edwards [30], Bubeck et al. [31], Lindell and Whitney [44], Armas [55], Becker et al. [56], and Huang et al. [57], several control variables were considered. These variables reflect residents’ individual and family socio-economic characteristics (Table 4).

#### 2.2.2. The Models

Residents’ evacuation willingness and relocation willingness in earthquake-stricken areas, the dependent variables of this study, were ordered multi-classification variables. Thus, ordinal logistic regression models were used in this study. The formula for the model was as follows:

$$\begin{array}{l} \mathrm{Logit}\left(Y_{1}\right)=\alpha_{0}+\alpha_{1}\times RP_{i}+\alpha_{2}\times SOP_{i}+\alpha_{3}\times RP_{i}\times SOP_{i}+\alpha_{4}\times {Control}_{i}+\varepsilon_{i}\\ \mathrm{Logit}\left(Y_{2}\right)=\beta_{0}+\beta_{1}\times RP_{i}+\beta_{2}\times SOP_{i}+\beta_{3}\times RP_{i}\times SOP_{i}+\beta_{4}\times {Control}_{i}+\sigma_{i} \end{array}$$

where, $Y_{1}$ and $Y_{2}$ refer to the willingness of residents to evacuate and relocate, respectively; $RP_{i}$ and $SOP_{i}$ are the core independent variables of the model, respectively representing residents’ disaster risk perception and sense of place; $\alpha_{0}$, $\alpha_{1}$, $\alpha_{2}$, $\alpha_{3}$, $\alpha_{4}$, $\beta_{0}$, $\beta_{1}$, $\beta_{2}$, $\beta_{3}$, and $\beta_{4}$ represent the model parameters to be estimated respectively; $\varepsilon_{i}$ and $\sigma_{i}$ represents the residual term. It should be noted that before constructing this interaction item ($RP_{i}\times SOP_{i}$), the two variables in the interaction item were centralized. Analysis of the models in this study was performed using Stata 11.0 (StataCorp. LLC, College Station, TX, USA).

#### 2.2.3. Theoretical Analysis and Research Hypotheses

Evacuation willingness and relocation willingness of residents in disaster threat areas, and their driving mechanisms, have been a research focus in the field of environmental psychology for some time. The existing literature indicates that disaster risk perception is one of the important factors affecting residents’ evacuation willingness and relocation willingness. Although there is variation in the research findings related to different types of disasters and different regions, overall, the literature appears to indicate that the possibility of disaster occurrence and the severity of disaster occurrence are significantly positively correlated with residents’ evacuation willingness and relocation willingness (e.g., [24,46]). At the same time, in the face of the threat of disasters, residents’ sense of place can also affect their behavioral decisions. For example, Xu et al. [24] found that place identity and place dependence were significantly negatively related to residents’ relocation willingness. Temporary evacuation and permanent relocation are two different situations. Temporary evacuation means that residents return home after the disaster. In this case, residents’ sense of place may not change much, and they may also evacuate quickly when receiving an earthquake warning due to consideration of the safety of life and property. However, permanent relocation is different. After arriving at their new residence, the resident must further adapt to the environment and reconstruct their sense of place. Further, among residents who live in disaster threat areas all year round, a sense of place may increase or decrease their disaster risk perception. For instance, residents with a particularly strong sense of place may have decreased perceptions of the possibility and severity of earthquake occurrence due to survivor bias; thus, they would be unwilling to evacuate and relocate. On the other hand, residents with a particularly weak sense of place may have increased perceptions of the possibility and severity of earthquake occurrence, leading to a strong willingness to evacuate and relocate.

**Figure 2:**
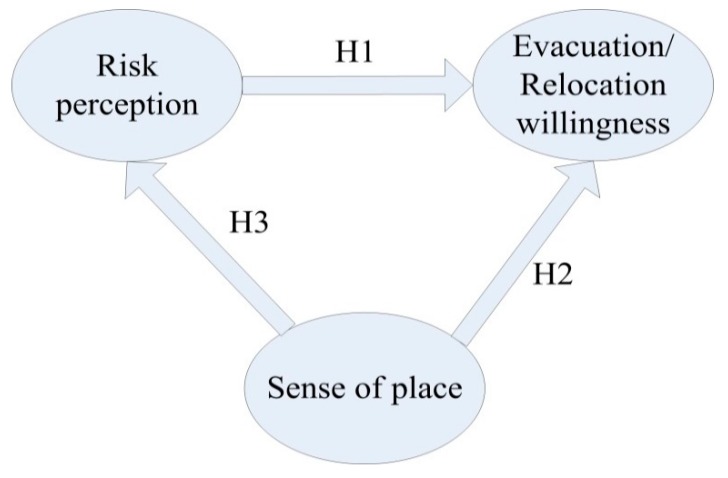
Research hypothesis framework.

Based on the above reasoning, this study constructed a research framework for residents’ disaster risk perception, sense of place, evacuation willingness, and relocation willingness (Figure 2). The following research hypotheses were proposed:

**Hypotheses** **1** **(H1).** *The two dimensions of disaster risk perception (the possibility of disaster occurrence and the severity of disaster occurrence) would be significantly positively correlated with residents’ evacuation willingness and relocation willingness.*

**Hypotheses** **2** **(H2).** *The three dimensions of sense of place would be significantly positively related to residents’ evacuation willingness but significantly negatively correlated with residents’ relocation willingness.*

**Hypotheses** **3** **(H3).** *Sense of place can increase or decrease residents’ disaster risk perceptions, thereby significantly affecting residents’ evacuation willingness and relocation willingness.*

## 3. Results

### 3.1. Descriptive Statistics

#### 3.1.1. Residents’ Evacuation Willingness and Relocation Willingness

**Figure 3:**
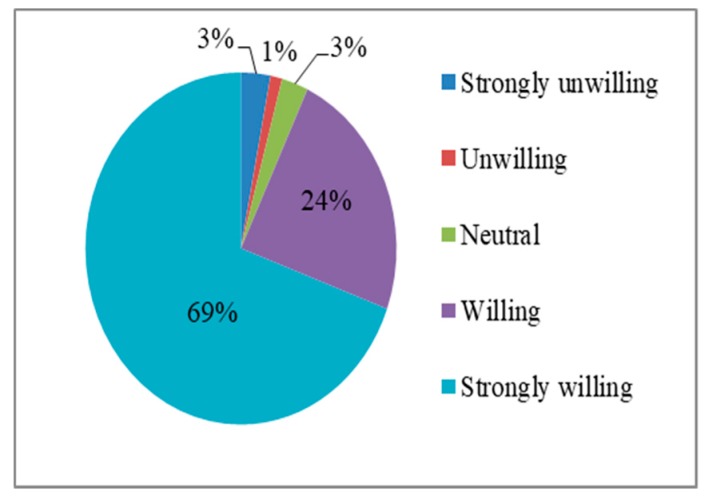
Frequency distribution map of residents’ evacuation willingness.

**Figure 4:**
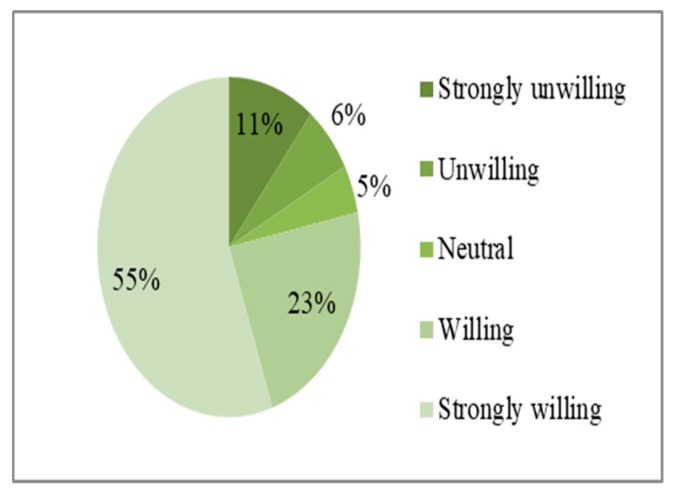
Frequency distribution map of residents’ relocation willingness.

Figure 3 and Figure 4 show the frequency distributions for residents’ evacuation willingness and relocation willingness variables, respectively. When facing the threat of earthquake disasters, residents have a strong willingness to evacuate and relocate. Among the 327 sample rural households, 69% and 55% of households were very willing to evacuate and relocate, respectively, and 24% and 23% of households were willing to evacuate and relocate, respectively. Relatively few individuals had neutral attitudes; 3% and 5% of rural households had neutral attitudes towards evacuation and relocation, respectively. Only 3% and 11% of residents were very unwilling to evacuate and relocate, respectively, and only 1% and 6% of rural households were unwilling to evacuate and relocate, respectively.

#### 3.1.2. Residents’ Disaster Risk Perceptions and Sense of Place

As shown in Table 4, the average scores for all dimensions of residents’ disaster risk perception and sense of place were above 60, indicating a relatively balanced and stable state. Further, the difference in the degree of dispersion was very small, and the standard deviations fluctuated between 6 and 9.

#### 3.1.3. Residents’ Individual and Family Socio-Economic Characteristics

As shown in Table 4, 46% of the respondents were women. The average age of the respondents was 53.44 years, while the average years of education were 6.29 years. Residents’ average time in their residence was 41.71 years. In total, 82% and 57% of the respondents were Han people and farmers, respectively. Moreover, 89% of rural households believed that the experience of major earthquake disasters is very serious. With regard to residents’ family socio-economic characteristics, the average annual family cash income was 66,238.94 Yuan. In total, 48% and 24% of families contained elderly people and children, respectively, while 48% of rural households had concrete houses. Furthermore, 44%, 16%, and 40% of rural households’ information primarily came from the government, relatives and friends, and the media, respectively.

### 3.2. Model Results

Table 5 shows the correlation coefficient matrix of the model variables. All correlation coefficients between variables were less than 0.80, indicating no serious multicollinearity between the independent variables of the models. Table 6 shows the regression results for residents’ disaster risk perception, sense of place, and evacuation willingness. Model 1 includes only the three dimensions of sense of place, Model 2 includes only the two dimensions of disaster risk perception, while Model 3 includes control variables based on Model 1 and Model 2. Model 4 includes the interaction term on the basis of Model 3. The interaction terms were constructed from the sense of place and disaster risk perception variables that were significantly correlated in Model 3. The overall significance test statistics for all models (Wald χ^2^) indicate that all models were significant at least at the 0.1 level. Thus, subsequent analyses were conducted. The final model results are interpreted in accordance with Model 4.

As shown in Table 6, only place dependence and the severity of disaster occurrence were significantly positively correlated with residents’ evacuation willingness, and the results are robust. Specifically, when everything else remains constant, every one-unit increase in place dependence and severity corresponds to increases in the odds of willingness to evacuate by a factor of 0.042 and 0.051, respectively. Meanwhile, as shown in Model 4, the interaction term between place dependence and the severity of disaster occurrence was significantly negatively related to residents’ evacuation willingness, indicating that place dependence can reduce the perception of the severity of disaster risks, thereby affecting evacuation willingness. Specifically, every one-unit increase in place dependence × severity corresponds to a decrease in the odds of willingness to evacuate by a factor of 0.004. Interestingly, none of the individual and family socio-economic variables were significantly correlated with residents’ evacuation willingness.

Table 7 shows the regression results of residents’ disaster risk perception, sense of place, and relocation willingness. The construction of Models 5–7 was similar to that of Models 1–3. Model 8 includes only the interaction term between place dependence and the severity of disaster occurrence, Model 9 includes only the interaction term between place identity and the severity of disaster occurrence, whereas Model 10 includes the interaction terms in Model 8 and Model 9. Similar to the results in Table 6, all the models in Table 7 passed the overall tests of model significance.

As shown in Table 7, place identity was significantly negatively correlated with residents’ relocation willingness, while place dependence and the severity of disaster occurrence were significantly positively related to residents’ relocation willingness. Specifically, when everything else remains constant, every one-unit increase in place identity corresponds to a decrease in the odds of willingness to relocate by a factor of 0.034, while every one-unit increase in place dependence and severity corresponds to increases in the odds of willingness to relocate by factors of 0.041 and 0.028, respectively (Model 7). At the same time, the interaction term between place dependence and the severity of disaster occurrence as well as the interaction term between place identity and the severity of disaster occurrence were significantly negatively correlated with residents’ relocation willingness, suggesting that place dependence and place identity can reduce the perception of the severity of disaster risks, thereby affecting relocation willingness. Specifically, every one-unit increase in place dependence × severity and identity × severity corresponds to decreases in the odds of willingness to relocate by factors of 0.003 and 0.003 (Model 8 and Model 9), respectively. Further, among all the control variables, only the severity of disaster experience was significantly positively related to residents’ relocation willingness. Specifically, every one-unit increase in experience corresponds to an increase in the odds of willingness to relocate by a factor of 0324 (Model 9). There were no other significant correlations between the control variables and residents’ relocation willingness.

## 4. Discussion

This study supplements the existing literature in the following ways. First, this study investigated rural households from the counties of the earthquake-stricken areas of Wenchuan Earthquake and Lushan Earthquake in Sichuan Province, China. These regions and households are extremely vulnerable to earthquake disasters, and this study further expands the scope of the research objects. Second, this study systematically analyzed residents’ disaster risk perceptions, sense of place, evacuation willingness, and relocation willingness and constructed econometric models to explore the correlations between these variables, focusing on the influence of the interaction between residents’ sense of place and disaster risk perceptions on residents’ evacuation willingness and relocation willingness. The introduction of relocation willingness and the interaction term between residents’ sense of place and disaster risk perception further widens the scope of the existing research.

In partial support of research hypothesis H1, this study found that disaster risk perception of residents in earthquake-stricken areas is an important factor affecting their evacuation willingness and relocation willingness. However, the results of this study are inconsistent with the findings of Xu et al. [24] and Mcneill et al. [46], who found that the possibility of disaster occurrence and the severity of disaster occurrence were significantly positively related to residents’ disaster avoidance behavior. In contrast, the current study found that the possibility of disaster occurrence was not significantly correlated with residents’ evacuation willingness and relocation willingness, while the severity of disaster occurrence was significantly positively related to residents’ evacuation willingness and relocation willingness. The possible reasons for the difference in results are the low frequency and the high severity of earthquake disasters. Residents believe that there have been several great earthquakes in the region in recent years, and the possibility of major earthquake reoccurrence in the next 10 years or even longer is perceived to be relatively low. However, the great earthquake disasters that have occurred have been rather catastrophic.

In partial support of research hypothesis H2, this study found that sense of place is also an important factor influencing residents’ evacuation willingness and relocation willingness. Unlike the study by Xu et al. [24] and Paton [44], the findings of Xu et al. [24] indicated that place identity and place dependence are significantly negatively correlated with residents’ relocation willingness. In contrast, this study found that place dependence was significantly positively related to residents’ evacuation willingness and relocation willingness, whereas place identity was only significantly negatively correlated with relocation willingness. The varied findings could be related to the different concepts of evacuation and relocation (which are systematically elaborated on in the theoretical analysis and hypothesis section of this paper).

Finally, to address research hypothesis H3, this study examined correlations between the sense of place and disaster risk perception interaction term and residents’ evacuation willingness and relocation willingness. The results indicated that the interaction term between place dependence and the severity of disaster occurrence was significantly negatively related to residents’ evacuation willingness and relocation willingness, and the interaction term between place identity and the severity of disaster occurrence was only significantly negatively correlated with residents’ relocation willingness. This further verifies that some dimensions of residents’ sense of place can reduce the perception of the severity of disaster occurrence, thereby affecting residents’ evacuation willingness and relocation willingness. These findings are consistent with research hypothesis H3.

Interestingly, for earthquake catastrophes in this study, the severity of earthquake disaster experience was significantly positively correlated with the residents’ relocation willingness, while none of the control variables related to residents’ individual and family socio-economic characteristics were significantly related to evacuation willingness and relocation willingness. This is in contrast to the results of many similar studies. For instance, Miceli et al. [58] found that gender was significantly positively correlated with flood mitigation behavior, Botzen and van den Berg [59] found that gender was significantly negatively related to flood mitigation behavior, while Lindell and Hwang [60] and Stein et al. [61] found no significant correlation between gender and disaster mitigation behavior. It is possible that the damage caused by the Wenchuan Earthquake and Lushan Earthquake was so serious (both earthquakes were above magnitude 7) that it left an indelible impression on the residents, such that residents have a strong willingness to evacuate and relocate as soon as they hear that the earthquake is about to happen, regardless of the household’s individual and family socio-economic characteristics. Residents have a clear understanding of the severity of earthquake disasters, and thus, most residents tend to report that they would relocate in an earthquake disaster situation. On the other hand, evacuation can only temporarily guarantee the safety of life, and after the earthquake, residents who have evacuated have to return to their homes and continue to be threatened by earthquake disasters. Therefore, in this study, the severity of earthquake disasters was not significantly correlated with residents’ evacuation willingness.

In addition to supplementing the existing literature on disaster risk management, these findings have several policy implications. Based on the current findings, when policymakers consider mobilizing the evacuation or relocation of residents in earthquake-stricken areas, it would be necessary to enhance residents’ scientific understanding of disasters, treat residents with affection, and motivate residents with reason. At the same time, after the relocation of residents, there is a need to facilitate residents’ sense of place in their new location and help them to overcome the psychological impact of relocation from the perspective of sense of place.

## 5. Conclusions

Through the analysis above, the research mainly comes to the following three conclusions:

(1) Faced with the threat of earthquake disasters, residents have a strong willingness to evacuate and relocate. Specifically, 93% and 78% of the residents in the Wenchuan Earthquake and Lushan Earthquake areas were willing to evacuate and relocate, respectively, whereas 4% and 17% of the residents were unwilling to evacuate and relocate, respectively.

(2) Place dependence and the severity of disaster occurrence were significantly positively correlated with residents’ evacuation willingness, while the interaction term between place dependence and the severity of disaster occurrence was negatively related to residents’ evacuation willingness. Specifically, when everything else remains constant, every one-unit increase in place dependence and severity corresponds to increases in the odds of willingness to evacuate by factors of 0.042 and 0.051, respectively; every one-unit increase in place dependence × severity corresponds to a decrease in the odds of willingness to evacuation by a factor of 0.004.

(3) Place identity was significantly negatively correlated with residents’ relocation willingness, while place dependence and severity of disaster occurrence were positively related to residents’ relocation willingness. The interaction term between place dependence and the severity of disaster occurrence as well as the interaction term between place identity and severity of disaster occurrence were significantly negatively correlated with residents’ relocation willingness. Specifically, every one-unit increase in place identity corresponds to a decrease in the odds of willingness to relocate by a factor of 0.034, while every one-unit increase in place dependence and severity corresponds to increases in the odds of willingness to relocate by factors of 0.041 and 0.028, respectively, and every one-unit increase in place dependence × severity and place identity × severity corresponds to decreases in the odds of willingness to relocate by factors of 0.003 and 0.003, respectively.

## Figures and Tables

**Table 1:** Sense of place measurement.

Entry Code	Dimension	Item ^a^	Mean	SD ^b^
PI1	Place identity	I do not want to move out of here because I’m used to the lifestyle here.	4.06	1.06
PI2		I am afraid of earthquakes, but I still don’t want to move away from here because my roots are here.	3.91	1.17
PI3		I’ve never thought that I’d move out of the village and live in other places.	3.37	1.51
PA1	Place attachment	I am more comfortable in the village, and I can do whatever I want.	4.41	0.81
PA2		My love for this village is deeper than that for anywhere else.	4.02	1.03
PA3		Unless going out to do some errands, I usually prefer to stay in the village.	4.31	0.90
PD1	Place dependence	If possible, I would like to participate in the decision-making of public affairs of the village.	3.87	1.19
PD2		I am very familiar with all the things in the village, and have a sense of security and control in the village.	4.21	0.87
PD3		I have a certain say in the public affairs of the village, and my wills are respected.	3.41	1.20

Note: ^a^ 1 = totally disagree, 2 = disagree, 3 = neutral, 4 = agree, 5 = totally agree; ^b^ SD = Standard deviation.

**Table 2:** Component matrixes for respective components of sense of place after rotation.

Items	Components		
	Place Identity	Place Attachment	Place Dependence
PI1	0.85	0.16	0.07
PI2	0.83	0.14	0.14
PI3	0.65	0.28	0.05
PA1	0.07	0.83	0.07
PA2	0.28	0.78	0.20
PA3	0.39	0.70	0.10
PD1	−0.01	−0.06	0.82
PD2	0.18	0.14	0.72
PD3	0.10	0.35	0.65
Eigenvalue	3.43	1.37	1.01
Explained variance	23.22%	22.47%	18.81%
Cumulative variance	23.22%	45.69%	64.50%
Cronbach’s α	0.71	0.76	0.61

**Table 3:** Earthquake disaster risk perception measurement.

Entry Code	Dimension	Item ^a^	Mean	SD ^b^
A1	Possibility	In the next 10 years, there will be earthquakes near my home.	2.83	1.12
A2		I always feel that an earthquake will come one day.	3.08	1.32
A3		In recent years, the signs of earthquake disaster occurrence have become more and more obvious.	3.18	1.35
A4	Severity	I’m worried about the impact of an earthquake on the village and the Family.	4.19	1.12
A5		In the next 10 years, if an earthquake occurs, your and your families’ lives will be affected.	3.35	1.31
A6		If an earthquake occurs, the production and life of the villagers will be seriously affected.	4.16	1.06

Note: a 1 = totally disagree, 2 = disagree, 3 = neutral, 4 = agree, 5 = totally agree; b SD = Standard Deviation.

**Table 4:** Definition and descriptive statistics of the variables in the model.

Category	Variable	Definition and Measure	Mean	SD ^d^
Dependent variable	Evacuation	Are rural households willing to evacuate when someone in the same village evacuates ^a^	4.55	0.86
	Relocation	Are rural households willing to relocate when someone in the same village relocates? ^a^	4.06	1.35
Risk perception	Possibility	Scores for perception of the possibility of an earthquake (1–100)	59.97	8.85
	Severity	Scores for perception of the severity of an earthquake (1–100)	60.00	8.28
Sense of place	Place identity	Scores for place identity of farming households (1–100)	60.00	7.66
	Place attachment	Scores for place attachent of farming households (1–100)	60.00	6.12
	Place dependence	Scores for place dependence of farming households (1–100)	60.00	7.89
Individual characteristics	Gender	Responder gender (0 = male, 1 = female)	0.46	0.50
	Age	Responder age (year)	53.44	13.40
	Education	Years of education (year)	6.29	3.70
	Residence	Length of residence of responder (year)	41.71	19.78
	Nationality	Responder nationality (0 = other, 1 = Han)	0.82	0.39
	Occupation	Responder occupation (0 = other, 1 = Farmer)	0.57	0.50
	Experience	The severity of residents’ disaster experience ^b^	4.56	0.76
Household characteristics	Income	Total annual cash income of rural households (Yuan ^c^)	66,238.94	72,237.87
	Old	Whether the resident family comprises individuals over 64 years of age (0 = no, 1 = yes)	0.48	0.50
	Child	Whether the resident family has a child below 6 years of age (0 = no, 1 = yes)	0.24	0.43
	House	Whether the house is a concrete structure (0 = no, 1 = yes)	0.48	0.50
	Information	Main sources of disaster information (1 = government, 2 = relatives, 3 = media)	1.97	0.92

Note: a 1 = strongly unwilling, 2 = unwilling, 3 = neutral, 4 = willing, 5 = strong willing; b 1 = not very serious, 2 = not serious, 3 = general, 4 = serious, 5 = very serious; c 1 USD = 6.88 Yuan (at the time of the study); d SD = Standard deviation.

**Table 5:** Correlation coefficient matrix of model variables.

**Variables**	**1**	**2**	**3**	**4**	**5**	**6**	**7**	**8**	**9**	**10**	**11**	**12**
1. Evacuation	1											
2. Relocation	0.417 ***	1										
3. Place identity	0.018	−0.137 **	1									
4. Place attachment	−0.006	−0.026	0.000	1								
5. Place dependence	0.126 **	0.115 **	0.000	0	1							
6. Possibility	0.010	0.007	−0.108 *	0.074	−0.056	1						
7. Severity	0.153 ***	0.110 **	−0.156 ***	−0.012	−0.043	0	1					
8. Gender	0.006	−0.013	−0.031	−0.053	−0.132 **	−0.043	0.069	1				
9. Age	−0.069	−0.023	0.086	0.162 ***	−0.050	0.130 **	−0.076	−0.212 ***	1			
10. Education	−0.012	0.007	−0.035	−0.212 ***	0.168 ***	−0.196 ***	−0.072	−0.136 **	−0.496 ***	1		
11. Experience	0.079	0.133 **	−0.059	0.146 ***	0.078	0.059	0.227 ***	0.000	−0.017	−0.0440	1	
12. Nationality	−0.014	−0.033	−0.008	0.009	−0.010	−0.088	−0.044	−0.060	−0.002	0.177 ***	−0.040	1
13. Occupation	0.040	0.032	−0.032	0.057	0.017	0.101 *	0.033	0.102 *	0.271 ***	−0.371 ***	0.026	−0.050
14. Residence	−0.060	0.008	0.080	0.142 **	0.079	0.166 ***	−0.035	−0.268 ***	0.517 ***	−0.343 ***	−0.031	−0.036
15. Old	−0.064	−0.033	0.144 ***	0.069	0.020	−0.058	0.031	−0.135 **	0.272 ***	−0.185 ***	−0.024	0.072
16. Child	−0.037	0.023	−0.043	−0.047	−0.048	0.066	0.043	0.079	−0.178 ***	0.176 ***	0.005	0.079
17. House	−0.031	−0.114 **	0.008	−0.047	0.027	−0.091 *	−0.144 ***	0.043	−0.132 **	0.261 ***	0.005	0.117 **
18. ln(income)	0.077	0.021	0.030	−0.023	0.165 ***	−0.166 ***	0.008	0.038	−0.226 ***	0.258 ***	0.133 **	0.039
19. Government	0.052	0.067	−0.012	0.028	0.172 ***	0.068	0.059	−0.013	0.003	0.062	0.135 **	−0.051
20. Friends	−0.074	−0.081	0.052	0.003	−0.225 ***	0.052	0.032	0.084	0.052	−0.188 ***	−0.047	0.030
21. Media	0.002	−0.008	−0.027	−0.031	−0.006	−0.107 *	−0.084	−0.049	−0.042	0.077	−0.101 *	0.029
**Variables**	**13**	**14**		**15**	**16**	**17**		**18**	**19**	**20**		**21**
13. Occupation	1											
14. Residence	0.161 ***	1										
15. Old	0.076	0.231 ***		1								
16. Child	−0.078	−0.148 ***		−0.074	1							
17. House	−0.152 ***	−0.231 ***		−0.158 ***	0.087	1						
18. ln(income)	−0.263 ***	−0.129 **		−0.145 ***	0.206 ***	0.281 ***		1				
19. Government	−0.115 **	0.049		−0.014	−0.008	0.004		0.055	1			
20. Friends	0.019	0.046		0.082	−0.050	−0.167 ***		−0.123 **	−0.383 ***	1		
21. Media	0.102 *	−0.084		−0.047	0.045	0.120 **		0.036	−0.725 ***	−0.358 ***		1

Note: *** *p* < 0.1; ** *p* < 0.05; * *p* < 0.1; 1–21 respectively represent Evacuation to Media. Wherein, Government, Friends, and Media respectively refer to the three channels of information sources of residents’ earthquake disaster.

**Table 6:** Regression results of residents’ disaster risk perception, sense of place, and evacuation willingness.

Variables	Model 1	Model 2	Model 3	Model 4
Place identity	0.006		0.016	0.014
	(0.017)		(0.016)	(0.016)
Place attachment	0.024		0.022	0.024
	(0.020)		(0.021)	(0.020)
Place dependence	0.040 ***		0.048 ***	0.042 **
	(0.015)		(0.017)	(0.016)
Possibility		−0.015	−0.014	−0.014
		(0.013)	(0.014)	(0.014)
Severity		0.051 ***	0.051 ***	0.051 ***
		(0.016)	(0.018)	(0.018)
Place dependence × Severity				−0.004 **
				(0.002)
Gender			0.204	0.222
			(0.311)	(0.316)
Age			0.008	0.005
			(0.014)	(0.014)
Education			−0.022	−0.032
			(0.049)	(0.049)
Experience			0.265	0.214
			(0.168)	(0.166)
Nationality			0.336	0.343
			(0.307)	(0.304)
Occupation			0.159	0.130
			(0.277)	(0.271)
Residence			−0.008	−0.006
			(0.009)	(0.009)
Old			−0.124	−0.125
			(0.268)	(0.270)
Child			−0.045	−0.016
			(0.310)	(0.311)
House			−0.047	0.008
			(0.278)	(0.276)
Information2 ^a^			0.228	0.169
			(0.436)	(0.421)
Information3 ^a^			0.144	0.199
			(0.274)	(0.277)
Ln(income)			0.051	0.046
			(0.150)	(0.150)
Wald chi2(χ^2^)	8.44	11.29	27.33	31.03
Prob > chi2(χ^2^)	0.037	0.003	0.072	0.040
Pseudo R^2^	0.015	0.025	0.057	0.065

Note: Robust standard errors in parentheses; *** *p* < 0.01, ** *p* < 0.05, * *p* < 0.1; ^a^ Information was sourced from the government as a control group.

**Table 7:** Regression results of residents’ disaster risk perception, sense of place and relocation willingness.

Variables	Model 5	Model 6	Model 7	Model 8	Model 9	Model 10
Place identity	−0.034 **		−0.034 **	−0.034 **	−0.039 **	−0.039 **
	(0.016)		(0.016)	(0.016)	(0.018)	(0.017)
Place attachment	0.001		−0.006	−0.004	−0.007	−0.006
	(0.021)		(0.022)	(0.022)	(0.021)	(0.021)
Place dependence	0.042 ***		0.041 ***	0.037 **	0.041 ***	0.037 **
	(0.014)		(0.015)	(0.015)	(0.015)	(0.015)
Possibility		−0.004	−0.010	−0.010	−0.010	−0.010
		(0.014)	(0.015)	(0.015)	(0.014)	(0.015)
Severity		0.035 **	0.028 *	0.028 *	0.028 *	0.027 *
		(0.014)	(0.015)	(0.015)	(0.014)	(0.015)
Place dependence × Severity				−0.003 *		−0.002
				(0.001)		(0.002)
Place identity × Severity					−0.003 *	−0.003 *
					(0.001)	(0.001)
Gender			0.115	0.129	0.124	0.136
			(0.259)	(0.260)	(0.258)	(0.258)
Age			0.013	0.011	0.012	0.011
			(0.010)	(0.010)	(0.010)	(0.010)
Education			0.019	0.013	0.020	0.013
			(0.040)	(0.040)	(0.040)	(0.040)
Experience			0.323 **	0.301 **	0.324 **	0.302 **
			(0.138)	(0.136)	(0.137)	(0.136)
Nationality			0.080	0.081	0.120	0.119
			(0.279)	(0.276)	(0.281)	(0.277)
Occupation			0.163	0.139	0.128	0.108
			(0.273)	(0.273)	(0.271)	(0.271)
Residence			0.000	0.001	−0.000	0.001
			(0.008)	(0.008)	(0.007)	(0.007)
Old			−0.104	−0.095	−0.067	−0.060
			(0.231)	(0.232)	(0.230)	(0.231)
Child			0.304	0.328	0.263	0.286
			(0.290)	(0.291)	(0.293)	(0.293)
House			−0.320	−0.293	−0.331	−0.302
			(0.250)	(0.252)	(0.252)	(0.253)
Information2 ^a^			−0.235	−0.266	−0.219	−0.249
			(0.334)	(0.332)	(0.332)	(0.330)
Information3 ^a^			0.071	0.113	0.108	0.143
			(0.245)	(0.248)	(0.248)	(0.249)
Ln(income)			0.016	0.013	0.035	0.033
			(0.134)	(0.135)	(0.135)	(0.136)
Wald chi2(χ^2^)	15.39	6.49	28.84	34.73	29.38	31.18
Prob > chi2(χ^2^)	0.002	0.039	0.050	0.015	0.060	0.053
Pseudo R ^2^	0.018	0.009	0.041	0.044	0.045	0.047

Note: All footnotes are consistent with Table 6.
